# Influence of Chronobiology on the Nanoparticle-Mediated Drug Uptake into the Brain

**DOI:** 10.3390/pharmaceutics7010003

**Published:** 2015-02-03

**Authors:** Jörg Kreuter

**Affiliations:** Institut für Pharmazeutische Technologie, Goethe-Universität Frankfurt, Max-von-Laue-Str. 9, Frankfurt D-60439, Germany; E-Mail: Kreuter@em.uni-frankfurt.de; Tel.: +49-69-9686-6000

**Keywords:** nanoparticles, blood–brain barrier, chronobiology, dalargin, day time dependent transport, neurosciences

## Abstract

Little attention so-far has been paid to the influence of chronobiology on the processes of nanoparticle uptake and transport into the brain, even though this transport appears to be chronobiologically controlled to a significant degree. Nanoparticles with specific surface properties enable the transport across the blood–brain barrier of many drugs that normally cannot cross this barrier. A clear dependence of the central antinociceptive (analgesic) effects of a nanoparticle-bound model drug,* i.e.*, the hexapeptide dalargin, on the time of day was observable after intravenous injection in mice. In addition to the strongly enhanced antinociceptive effect due to the binding to the nanoparticles, the minima and maxima of the pain reaction with the nanoparticle-bound drug were shifted by almost half a day compared to the normal circadian nociception: The maximum in the pain reaction after i.v. injection of the nanoparticle-bound dalargin occurred during the later rest phase of the animals whereas the normal pain reaction and that of a dalargin solution was highest during the active phase of the mice in the night. This important shift could be caused by an enhanced endo- and exocytotic particulates transport activity of the brain capillary endothelial cells or within the brain during the rest phase.

## 1. Introduction

Drug delivery to the brain is restricted or for most drugs totally prevented by the blood–brain barrier (BBB) [1,2,3]. One possibility to overcome this barrier is a delivery of the drugs by binding them to biocompatible and rapidly biodegradable nanoparticles [4]. Materials for such particles include poly(alkyl cyanoacrylates), polylactides, and crosslinked human serum albumin (HSA). The mechanism of the transport across the BBB appears to be receptor-mediated endocytosis followed by transcytosis of the drug-loaded nanoparticles into the brain or by the release of the drugs within the endothelial cells [4,5,6,7]. In order to enable this receptor-mediated uptake the modification of the nanoparticle surface is necessary [4,5,6]. This can be achieved by overcoating of the nanoparticles with certain surfactants that adsorb specific apolipoproteins from the blood after intravenous injection [8,9] or by the covalent attachment of such apolipoproteins [10], transferrin [11], insulin [12], or other moieties to the particles that enable their interaction with the respective receptors on the brain capillary endothelial cells.

So-far, little attention has been paid to the influence of chronobiology on the processes of nanoparticle uptake and transport into the brain. However, these processes appear to be controlled by chronobiology to a significant extent [13]. Chronobiology,* i.e.*, biological rhythms, play a major role in the quantity and quality of a lot of disease states, biological transport processes, as well as drug actions [14]. Indeed, these rhythms cover nearly every division of time: oscillations of one per second (e.g., in the electroencephalogram), one per several seconds (e.g., respiratory rhythm, heart rate), one within 24 h (e.g., circadian rhythms), up to one per year (e.g., circannual rhythms) [15]. Blood pressure, heart rate, peripheral resistance, pressure and the release/activity of vasodilating hormones all display pronounced circadian variations [16]. The mammalian circadian clock is located in the neurons of suprachiasmatic nuclei (SCN) in the brain and in peripheral tissues [17,18]. At a cellular level large portions of cellular physiology - from transcription and translation to intracellular signaling cascades - can show daily variations in activity [19]. Numerous studies in animals, as well as clinical studies, have provided convincing evidence that the pharmacokinetics and/or the effects/side effects of drugs can be modified by the circadian time and/or the timing of drug administration within 24 h of a day [20]. A circadian phase dependency also is known for the perception of pain [21,22]. Rhythmicity also has been demonstrated for the concentrations of neurotransmitters involved in pain modulation. Moreover, opiate receptor binding in various regions of the rat brain was demonstrated to vary with circadian time [23].

## 2. Circadian Influence on the Pharmacological Activity of an Analgesic Hexapeptide Delivered by Nanoparticles across the Blood–Brain Barrier

**Figure 1:**
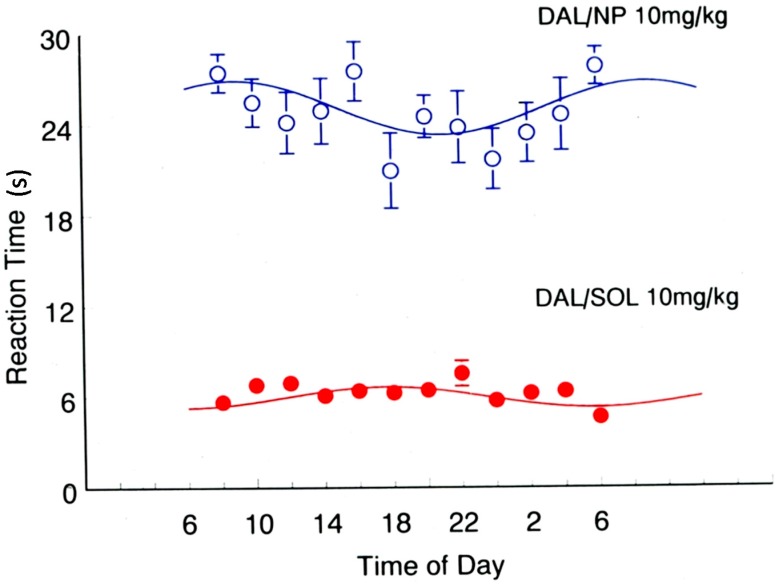
Dependence of the reaction times (s) in the hot-plate test 15 min after intravenous injection of 10 mg/kg of a dalargin solution (DAL/SOL) or of dalargin-coated nanoparticles (DAL/NP) on the time of day. Groups of 10–12 DAB/2 mice were tested every 2 h within 24 h; lights on from 07:00 to 19:00. Shown are mean values ± SEM; the solid line represents the cosine fit to the data. (Adapted with permission from [13]. Copyright 1999 Informa Healthcare).

**Figure 2:**
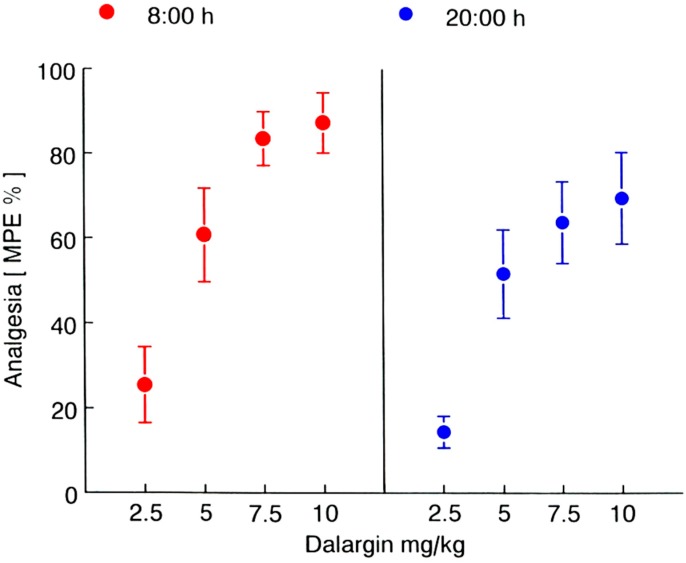
Dose-response effects of dalargin-coated nanoparticles. Response determined 15 min after intravenous injection (DAL/NP, 2.5–10 mg/kg) at either 08:00 or 20:00; lights on from 07:00 to 19:00. Shown are mean values ± SEM of the maximal possible effect (% MPE. Adapted with permission from [13]. Copyright 1999 Informa Healthcare).

Ramge* et al.* [13] addressed the problem of the influence of chronobiology on the drug transport across the BBB and investigated the fluctuations and the day-time dependence of the pain reactivity of mice using the tail-flick as well as the hot plate test models confirming earlier data of Frederickson* et al.* [24]: A maximal pain reaction indicated by a minimal reaction time was observed during the night time which represents the activity phase of the mice. The intravenous injection of a solution of the antinociceptive (analgesic) model drug dalargin did not change this pattern of the pain reaction (Figure 1, lower curves). Dalargin is a hexapeptide that being an artificial endorphin, has antinociceptive properties upon injection directly into the brain ventricles, but is not able to produce such effects after intravenous injection as it is unable to permeate the BBB [25]. However, as already shown earlier [26,27], the binding of dalargin to poly(butyl cyanoacrylate) and additional overcoating with polysorbate 80 (Tween^®^ 80) enabled the transport of this drug across the BBB into the brain and yielded a significant dose-dependent non-nociceptive effect after intravenous injection (Figure 1, upper curve and Figure 2). Without the polysorbate coating the drug-loaded particles cannot interact with the respective receptors and, therefore, also are not able to traverse the BBB [4,5,6,26,27]. However, as mentioned above, the coating with polysorbate 80 led to the adsorption of apolipoprotein E [9,28] which then interacted with the LRP1-receptors located on the brain capillary endothelial cells, followed by endocytosis and transcytosis of the nanoparticles across these cells [4,5,6,7,29,30].

This effect was strongly day time-dependent (Figure 1 and Figure 2). As a consequence, the maximal possible effect (MPE) increased to 90% in the morning (8:00) and to only 70% in the evening (20:00) at a dalargin dose of 10 mg/kg, again showing the influence of the circadian rhythm (Figure 2).

Even more interesting was the finding that whereas the pain reactions without drug and with dalargin solution were highest (shortest reaction times) during the active phase of the mice in the night, the maximum in the pain reaction after i.v. injection of the nanoparticle-bound dalargin occurred in the later day times resulting in a shift of the calculated circadian response curve of over 10 h, almost a half-day shift (Figure 1).

This result is very surprising. One would expect that most processes are more active during the activity phase of the animals. Cerebral blood flow, for instance, has been shown to also be circadian phase-dependent, being greater during the activity period of nocturnal animals [31]. As a consequence, a more efficient transport of the nanoparticles by the blood to the brain endothelium would be anticipated. Also the energy-dependent endocytosis and transcytosis processes also should be expected to be up-regulated and more operative, and hence the reaction time should be increased accordingly during this time. However, the opposite is the case, the reduction of the pain reaction time by the dalargin-loaded nanoparticles was 20% lower in the animals’ activity phase than in the rest phase, indicated by the observed lower MPE (Figure 2).

One possible explanation for this result could be that the quality or quantity of the interaction of dalargin on the level of the opioid receptors in the brain is somehow altered by the intrinsic properties of the nanoparticles causing a decelerated transport of the particles into or within the brain or a retarded release of dalargin. However, in all experiments with dalargin, loperamide, tubocurarine, or other drugs the response times where very short [4]. The antinociceptive responses displayed in Figure 1 and Figure 2, for instance, were recorded 15 min after intravenous injection. For this reason this possibility appears to be unlikely. Another possibility is that the chronobiological fluctuation of the expression in the quantity of the opioid receptor in various regions of the rat brain could affect the drug effect [23], regardless of intrinsic effect of the nanoparticles. In this case the observed 10 h shift in the minima and maxima caused by binding to the nanoparticles would be difficult to interpret as this fluctuation should be similar in both cases and only could be explained by an alteration of the drug disposition in the brain by the nanoparticles compared to free drug. Again, the rapid pharmacological response times requiring a fast release speak against this. It, therefore, appears to be most likely that during the rest phase of the animals the activity of certain energy-requiring transport processes such as endocytosis and transcytosis is upregulated. This is of course a speculative hypothesis that has to be further substantiated experimentally.

The above finding has very important and further reaching implications. It has to be noted that the difference between the day-time and night time nanoparticle-caused effects is considerable,* i.e.*, over 20%. This non-negligible difference demonstrates that the outcome of results concerning the drug transport across the BBB using nanoparticles significantly depend on the time of day of the experiments. Therefore, in comparing different experiments this fact has to be taken in consideration. Secondly, it should be kept in mind that these findings also transcend to the influence of chronobiology on the permeation process efficacy of other particulates across this barrier. This includes the transcellular lymphocyte migration through the brain endothelium [32] or the BBB crossing of endogenous particulates such as antibodies or of pathogens like viruses and bacteria that also may be day time-dependent and could be more active during the rest phase. The complexity and interactivity of these processes may make it difficult to elucidate the single contribution of these processes.

## 3. Conclusions

The pain reaction of mice was significantly influenced and altered in a dose-dependent fashion by the nanoparticle-mediated transport of an antinociceptive (analgesic) drug, dalargin, across the blood–brain barrier. The minima and maxima of the pain reaction with the nanoparticle-bound drug were shifted by almost half a day compared to the normal circadian nociception. The maximum in the pain reaction after i.v. injection of the nanoparticle-bound dalargin occurred during the later rest phase of the animals whereas the normal pain reaction was highest during the active phase of the mice in the night. This shift could be caused by an enhanced endo- and exocytotic nanoparticle transport activity of the brain capillary endothelial cells or within the brain during the rest phase.
